# Thicker Temporal Cortex Associates with a Developmental Trajectory for Psychopathic Traits in Adolescents

**DOI:** 10.1371/journal.pone.0127025

**Published:** 2015-05-27

**Authors:** Yaling Yang, Pan Wang, Laura A. Baker, Katherine L. Narr, Shantanu H. Joshi, George Hafzalla, Adrian Raine, Paul M. Thompson

**Affiliations:** 1 Department of Pediatrics, Children’s Hospital Los Angeles, University of Southern California, Los Angeles, California, United States of America; 2 Department of Psychology, University of Southern California, Los Angeles, California, United States of America; 3 Department of Neurology, David Geffen School of Medicine at UCLA, Los Angeles, California, United States of America; 4 Departments of Criminology, Psychiatry, and Psychology, University of Pennsylvania, Philadelphia, Pennsylvania, United States of America; 5 Departments of Neurology, Psychiatry, Radiology, Engineering, Pediatrics, and Ophthalmology, University of Southern California, Los Angeles, California, United States of America; Leibniz Institute for Neurobiology, GERMANY

## Abstract

Psychopathy is a clinical condition characterized by a failure in normal social interaction and morality. Recent studies have begun to reveal brain structural abnormalities associated with psychopathic tendencies in children. However, little is known about whether variations in brain morphology are linked to the developmental trajectory of psychopathic traits over time. In this study, structural magnetic resonance imaging (sMRI) data from 108 14-year-old adolescents with no history of substance abuse (54 males and 54 females) were examined to detect cortical thickness variations associated with psychopathic traits and individual rates of change in psychopathic traits from ages 9 to 18. We found cortical thickness abnormalities to correlate with psychopathic traits both cross-sectionally and longitudinally. Specifically, at age 14, higher psychopathic scores were correlated with thinner cortex in the middle frontal gyrus, particularly in females, and thicker cortex in the superior temporal gyrus, middle temporal gyrus, and parahippocampal gyrus, particularly in males. Longitudinally, individual rates of change in psychopathic tendency over time were correlated with thicker cortex in the superior temporal gyrus, middle temporal gyrus, inferior temporal gyrus, parahippocampal gyrus, and posterior cingulate gyrus, particularly in males. Findings suggest that abnormal cortical thickness may reflect a delay in brain maturation, resulting in disturbances in frontal and temporal functioning such as impulsivity, sensation-seeking, and emotional dysregulation in adolescents. Thus, findings provide initial evidence supporting that abnormal cortical thickness may serve as a biomarker for the development of psychopathic propensity in adolescents.

## Introduction

Psychopathy is a clinical condition that affects ~1% of the general population and is linked to antisocial and criminal behavior [1]. Characterized by a cluster of personality traits including impulsivity, marked sensation-seeking, superficial emotion, and blunted punishment sensitivity, the concept of psychopathy has been successfully extended to younger groups to describe and understand antisocial behavior and aggression in youth [2–4]. In general, behavioral, temperamental, and neurocognitive characteristics in children with psychopathic tendencies resemble those of adults with psychopathy [5]. Psychopathic traits in younger samples reliably predict the later development of delinquency, aggression, and antisocial personality disorder [6], suggesting a potential neurodevelopmental basis to psychopathy. However, there remains a need to identify neurobiological characteristics associated with progressive increases in psychopathic personality traits throughout development as it would provide the much needed empirical evidence to help developing more effective early intervention and prevention methods for psychopathy and related disorders [4].

On the basis of normal brain development, childhood and adolescence represent periods of extraordinary improvement in the efficiency in processing sensory, motor, cognitive, and emotional information. Complex behavioral milestones during this time period are linked to intricate brain maturational processes that are both regressive (synaptic pruning) and progressive (myelination). MRI evidence revealed that gray and white matter in the brain undergo extensive growth during early childhood, followed by a decrease in gray matter volume, particularly considerable thinning of the cortex, starting around puberty [7–9]. The process does not occur uniformly across the whole brain, but follows a pattern of maturation sequence from inferior/posterior to superior/anterior in general [8, 10]. This may explain the inconsistency among findings of neurobiological correlates of psychopathic traits between developmental populations with varied age ranges.

Over the last two decades, psychopathy in adults has been repeatedly linked to brain alterations, particularly lower gray matter volumes and abnormal cortical thinning, in the frontal and temporal cortex [11–16]. These findings are recently replicated in a study of an unusually large cohort of incarcerated adult males (N = 296), which show psychopathy scores correlating with lower gray matter volumes in the orbitofrontal cortex, parahippocampal cortex, temporal poles and the posterior cingulate cortex [17]. These findings of frontotemporal and paralimbic alterations are consistent with findings of impairments in social cognition, affect processing and regulating, and moral decision-making, often observed in psychopathic individuals [18–20].

Similar structural abnormalities have been linked to psychopathic-related traits in children and adolescents (see reviews by [21–23]), however results to date remain inconclusive. For example, two studies by De Brito and colleagues showed 10–13 year old boys with both conduct problems and psychopathic tendencies to have greater gray matter and lower white matter concentration in the posterior medial orbitofrontal and anterior cingulate cortex compared to typically developing boys [24, 25]. On the other hand, in a large sample of incarcerated adolescent males (N = 215), psychopathic traits were associated with lower gray matter volume in the orbitofrontal cortex, bilateral temporal poles, and posterior cingulate cortex[26]. In another study, Cope and the colleagues also observed similar effects in a small group of incarcerated adolescent females (N = 39) that lower gray matter volumes were associated with psychopathic traits in the orbitofrontal cortex, parahippocampal cortex, temporal poles and left hippocampus[27]. Findings suggest that although there are overlaps in abnormalities observed in adults and adolescents, the neural correlation of psychopathic traits in children may be more complex, involving both hypoplasia and hyperplasia.

In this report, we tested whether psychopathic tendency and its developmental trajectory from childhood to early adolescence can be linked to specific characteristics of cortical thickness. By using scores of psychopathic traits collected at up to four time points from ages 9–18 (ages 9–10, 11–13, 14–15, 16–18; see [28]) and structural MRI data collected at age 14 from a relatively large cohort of 108 adolescents, we sought to determine if abnormal cortical thickness is associated with (1) psychopathic tendency at the time of the scanning, and (2) individual rates of change in psychopathic traits over time. The current study also benefits from the assessment of a homogeneously-aged population, which limits confounds associated with age-related variations in brain structure that are typically encountered in developmental studies. Based on the existing literature[19, 20, 22], we hypothesize that frontotemporal abnormalities will be associated with not only concurrent psychopathic tendency, but also changes in psychopathic tendency from childhood to adolescence. Specifically, we expect abnormal cortical thinning in regions involved in impulse control, affect regulation, and moral decision-making, particularly the orbitofrontal cortex, middle frontal cortex, cingulate cortex, and the superior temporal gyrus to be associated with higher concurrent level of psychopathic tendency in adolescents. Furthermore, we anticipate that abnormal cortical thickness in the temporal lobe will be most strongly linked to changes in psychopathic tendency as increasing demands in social adjustment, empathy and interpersonal bonding during adolescence would require more than sufficient functioning of this region.

## Materials and Methods

### Subjects

The 108 14-year-old adolescents included in this study were drawn from participants in the University of Southern California (USC) Risk Factors for Antisocial Behavior Twin Study [28, 29]. Briefly, the USC Twin Study is a longitudinal study assessing the development of antisocial behaviors from childhood to young adulthood. The adolescents and their families were recruited from Los Angeles County through advertisements, schools, and mothers of twins clubs. The sample is representative of the ethnic and socioeconomic diversity of the greater Los Angeles areas. Specifically, the ethnicity breakdown of the sample was as follows: 36.7% Hispanic, 27.4% Caucasian, 14% Black, 4.4% Asian,. 16% Native American, and 17.3% mixed. Participants were excluded if they had a history of significant head injury (that involves a loss in consciousness), major neurological, psychiatric illness, or contraindication to scanning (Baker et al., 2013; Yang et al., 2012). Based on the self-report survey on the history of substance use, 3 subjects had tried marijuana (on average 2 times), 24 subjects had tried a sip of alcohol (on average 3 times), 5 subjects had tried cigarettes (on average 2 times), and none had tried other drugs at the time of the brain scan (age 14). Thus, no subject was excluded because of prior or current substance abuse. The sex distribution of this sample was exactly equal with 50% boys and 50% girls. The adolescents and their primary caregivers (mainly their biological mothers) participated in 6–8 hours of laboratory assessment at USC, including the one-hour scan. Caregivers were administered self-report questionnaires and interviewed about their children’s behaviors. Both parents and children gave written informed consent/assent prior to the study. The study was approved by both the USC and the CHLA Institutional Review Boards.

### Psychopathy measures and estimating individual rates of change over time

**Fig 1:**
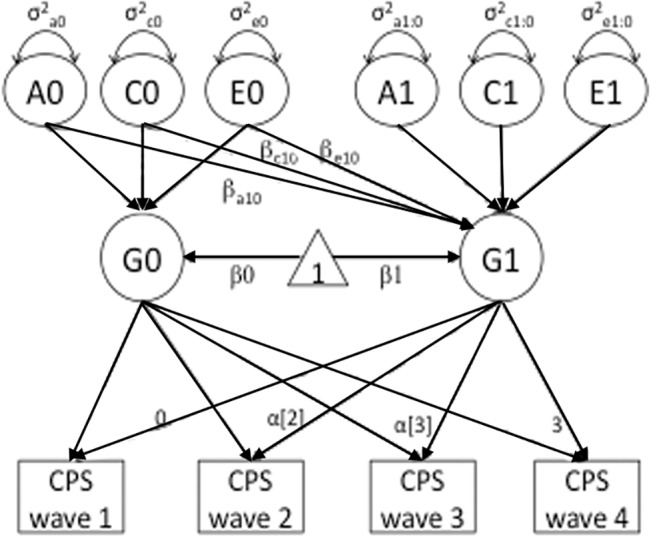
Mean CPS Scores for Total Sample, Males, and Females across Four Waves.

Children’s psychopathic traits were assessed using a slightly extended version of the Childhood Psychopathy Scale (CPS) [3, 30] as described previously [31, 32]. The CPS is a well-validated instrument for measuring psychopathic tendency in children and adolescents. The full scale contains 58 age-appropriate questions rated on a two-point scale (Yes = 2 or No = 1) and is composed of 14 subscales including assessments of glibness, grandiosity, boredom susceptibility, untruthfulness, manipulation, lack of guilt, poverty of affect, callousness, impulsiveness, parasitic lifestyle, behavioral dyscontrol, lack of planning, unreliability, and failure to accept responsibility. The CPS was administered to the caregivers in interview form and the score on all items was calculated to estimate the parent-reported CPS total score for each child at up to four waves: ages 9–10, 11–13, 14–15 (when brain scan was collected), and 16–18 (see Table 1 and Fig 1). Of all subjects, 36 were assessed at all four waves, 35 were assessed at 3 waves, and 37 were assessed at two waves.

**Table 1:** Mean CPS Score and the Slope Score (Rate of Change) for Total Sample, Males, and Females across Four Waves.

	Total Sample		Males		Females	
*CPS Total Score* ^a^ ^.^	Range	Mean (SD)	Range	Mean (SD)	Range	Mean (SD)
Wave 1 (ages 9–10)	3–42	12.26 (7.19)	3–42	14.20 (8.22)	3–26	10.33 (5.42)
Wave 2 (ages 11–13)	4–35	13.17 (7.77)	5–35	15.13 (8.69)	4–29	11.10 (6.14)
Wave 3 (ages 14–15)	2–33	12.18 (7.09)	3–33	13.96 (7.92)	2–31	10.39 (5.68)
Wave 4 (ages 16–18)	2–34	10.11 (6.47)	2–34	12.35 (7.97)	3–14	7.87 (3.41)
*CPS Slope Score*	Range	Mean (SD)	Range	Mean (SD)	Range	Mean (SD)
	-1.45–1.58	.18 (.50)	-1.45–1.58	.20 (.60)	-1.06–1.12	.16 (.40)

^a.^ CPS total score was obtained from 88 subjects (44 males and 44 females) at wave 1, 66 subjects (34 males and 32 females) at wave 2, 108 subjects (54 males and 54 females) at wave 3, and 46 subjects (23 males and 23 females) at wave 4.

**Fig 2:**
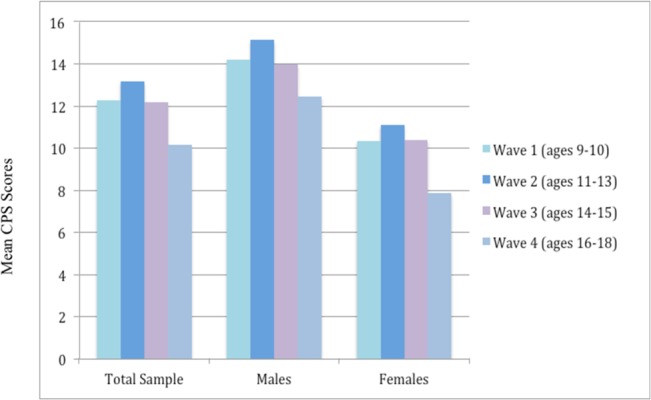
Biometric Latent Growth Model for Estimating Individual Rate of Change (Slope) for the CPS Scores. Here we adapted a classical biometric latent growth model—for psychopathic personality—to estimate the initial level (intercept; G_0_) and the rate of change (slope; G_1_) across four waves of assessment. We computed these from the intercept (β_0_) and age-based slope (β_1_) values, respectively. The triangle represents the unit constant where the one-headed arrow from the unit constant represents the mean; all paths that are not explicitly labeled in the diagram are set to one. The basis coefficients α_[t]_ are weights used to represent the function of time for the observations at wave 2 and wave 3.

In the present study, we focused on two developmental components of psychopathy: cross-sectional psychopathy scores (at the time of the brain scan) and the rate of change in psychopathy over time (i.e., the slope across waves). The individual rates of change over time (with direction) can be estimated with data from more than two time points and linked to brain morphometry to identify biomarkers for the trajectory of psychopathic tendency. To estimate the slope, an individual summary statistics is typically computed as the regression of outcomes Y_ij_ on time t_j_: Y_ij_ = β_0i_ + β _1i_t_j_ + ε_ij_. However, because our data involves related subjects, a more complex latent growth model (Fig 2), implemented using Mplus [33] [34, 35], was fitted to the scores from each available wave to obtain the intercept and change rate for the CPS scores for each individual. As illustrated in Fig 1, the initial level (intercept; G_0_) and the rate of change (slope; G_1_) across waves were determined by examining the intercept (β_0_) and age-based slope (β_1_) values for the CPS scores, and the basis coefficients α_[t]_ weights were used to represent the function of time for the observations at wave 2 and 3. Although not all subjects were assessed at all waves, this method allowed us to utilize all available data to identify neural correlates associated with the rate of change in psychopathic tendency from childhood to adolescence. It is worth mentioning that, of all 108 subjects, only 46 had psychopathy scores at age 16–18. Because this late adolescent period is an important age range for the manifestation of psychopathy, the reduced sample at this wave may have impacted our estimation of changes in psychopathic tendency over time.

### Image data acquisition and processing

All participants were scanned using a 3T Siemens Magnetom Trio whole-body scanner at the USC Dornsife Cognitive Neuroscience Imaging Center. 3D high-resolution T1-weighted images were acquired with a magnetization prepared rapid gradient echo (MP-RAGE) protocol with the following parameters: inversion time (TI)/ repetition time (TR)/ echo time (TE) = 800/ 2530/ 3.09 msec, slice thickness = 1 mm without gap, matrix = 256 x 256, and field of view (FOV) = 256 mm x 256 mm.

For each participant, cortical thickness was estimated using the FreeSurfer software v5.1.0 (13,14) at each vertex over the entire cortical surface, a process that has been validated against postmortem histological analysis [36] and manual measurements [37, 38]. In brief, FreeSurfer processing streams included skull-stripping, tissue segmentation, and spatial normalization of each image volume [39, 40]. To estimate cortical thickness, after intensity normalization, gray-white tissue segmentation was used to extract the pial and gray-white cortical surface. The pial and gray-white cortical surface of each subject was visually inspected and manually corrected to ensure accuracy [39, 40]. We then averaged the cortical surface shapes of all subjects included in this study to create a study-specific surface template and registered each individual cortical surface to the template to allow group analysis. To boost the signal to noise ratio, we applied a 25-mm full-width at half-maximum Gaussian surface-based smoothing kernel to the estimated thickness values [41]. Whole brain, total gray matter and total cortical white matter volume of each individual was also obtained using FreeSurfer, however none of these volumes were not significantly correlated with psychopathy scores at the time of the brain scan (Table 2).

**Table 2:** Pearson correlation coefficients (r) between total CPS scores at wave 3 (when brain scans were collected) and brain volumes.

	Mean CPS total scores at wave 3					
	Total Sample (N = 108)		Males (N = 54)		Females (N = 54)	
	r	P^a^	r	P^a^	r	P^a^
**Whole brain volume**	-.09	.34	-.16	.26	-.16	.26
**Total gray matter volume**	.16	.10	.9	.53	-.08	.55
**Total cortical white matter volume**	.09	.33	.11	.44	-.19	.18

^a^ The P values shown are two-tailed probability tests.

### Statistical analyses

To determine if cortical thickness variations across the whole brain are associated with (1) parent-reported psychopathy scores at the time of scanning, and (2) the estimated individual rates of change (i.e., slope) in psychopathy over time, vertex-by-vertex analysis was performed using a mixed effects regression model [39, 42, 43] implemented in the R statistical package [44], to allow the controlling for sex, whole brain volume, and subject relatedness. Relatedness in the twin pairs was accounted for in the covariance structure by allowing dependencies between the residuals in the regression analysis. In theory, this makes the results representative of those obtained from unrelated subjects. As comparisons were made at thousands of surface points, results reported here were thresholded using the standard False Discovery Rate (FDR) correction for multiple comparisons (*q*-value = 0.05) [45, 46]. Post-hoc analyses were conducted separating males and females to show gender-specific effects for each of the statistical analyses.

## Results

### Cortical thickness and concurrent psychopathy scores

**Fig 3:**
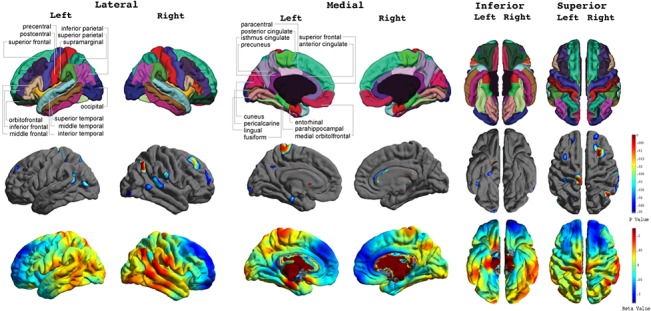
Concurrent Correlations between Cortical Thickness and Psychopathy Scores across the Total Sample. FDR-corrected P value maps (*top panel*) show significant correlations between cortical thickness and psychopathic traits at 14 years old. The corresponding uncorrected beta maps (*bottom panel*) show the direction of the correlations. Cold colors indicate negative correlations; hot colors indicate positive correlations.

As shown in Fig 3, cross-sectionally at age 14, elevated psychopathy scores correlated significantly with abnormal cortical thickness in several frontal and temporal regions. Specifically, higher psychopathy scores correlated with thinner cortex in the bilateral middle frontal cortex and thicker cortex in the bilateral middle temporal, left inferior temporal, right superior temporal, right supramarginal, left parahippocampal, left superior parietal, right inferior parietal, left precentral and postcentral, and bilateral lateral occipital gyri.

**Fig 4:**
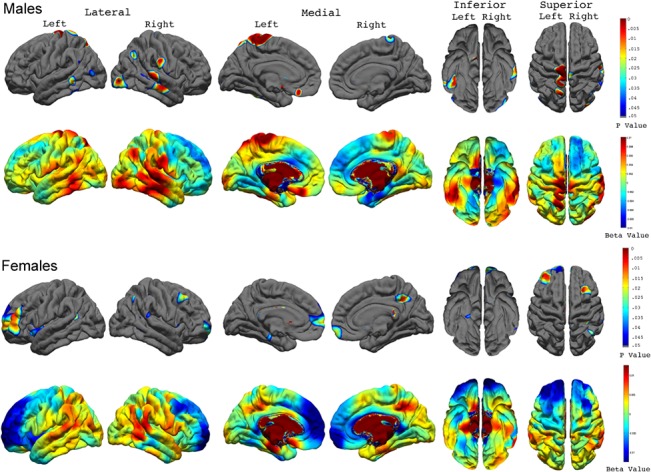
Concurrent Correlations between Cortical Thickness and Psychopathy Scores in Males and Females. FDR-corrected P value maps show significant correlations between cortical thickness and psychopathic traits at 14 years old in males and females with the corresponding uncorrected beta maps show the direction of the correlation.

In our post-hoc analyses for gender-specific effects (Fig 4), we found higher psychopathy scores in males to correlate significantly with thicker cortex in the bilateral middle temporal gyri, right superior temporal gyrus, left inferior temporal gyrus, left medial orbitofrontal gyrus, right supramarginal gyrus, bilateral paracentral gyri, left precuneus gyrus, bilateral precentral and postcentral gyri, and bilateral lateral occipital gyri. For females, higher psychopathy scores were correlated with thinner cortex in the bilateral middle frontal gyri, left superior frontal gyrus, right medial orbitofrontal gyrus, left insula, right isthmus cingulate gyrus, and thicker cortex in the bilateral superior temporal gyri.

### Cortical thickness and individual rates of change in psychopathy across waves

**Fig 5:**
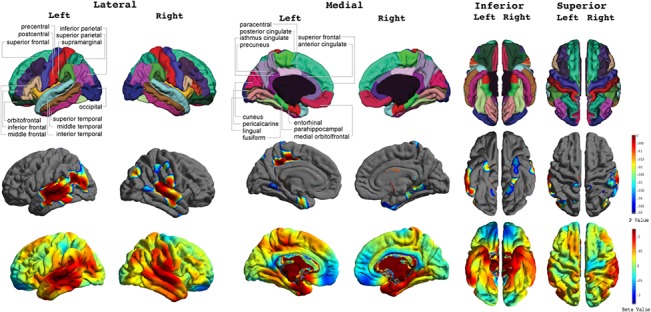
Correlations between Cortical Thickness and the Individual Rate of Change for Psychopathic Traits across the Total Sample. FDR-corrected P value maps (*top panel*) show significant correlations between cortical thickness and the individual rate of change for psychopathic traits over time. The corresponding uncorrected beta maps (*bottom panel*) show the direction of the correlations. Cold colors indicate negative correlations; hot colors indicate positive correlations.

Longitudinally, we found a significant relationship between abnormal cortical thickness and individual rates of change in psychopathy over time (Fig 5). Specifically, higher rates of change in psychopathic tendency (i.e., increases in psychopathy over time) correlated significantly with thicker cortex in the bilateral superior temporal, bilateral middle temporal, left inferior temporal, right supramarginal, bilateral inferior parietal, right superior parietal, left posterior and isthmus cingulate, bilateral parahippocampal, bilateral entorhinal, bilateral lingual, bilateral postcentral, and bilateral lateral occipital gyri.

**Fig 6:**
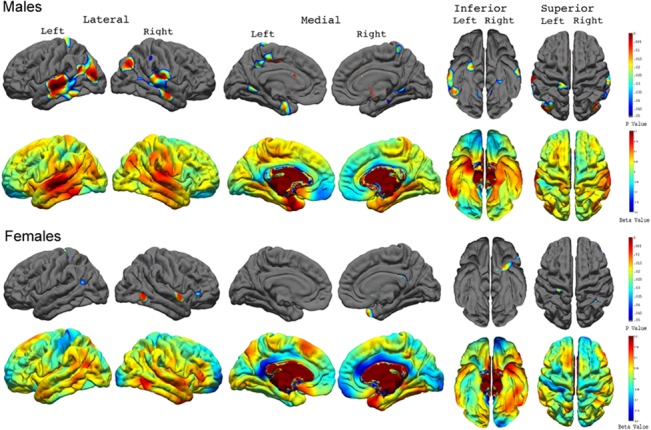
Correlations between Cortical Thickness and the Individual Rate of Change for Psychopathic Traits in Males and Females. FDR-corrected P value maps show significant correlations between cortical thickness and the individual rate of change for psychopathic traits over time in males and females with the corresponding uncorrected beta maps show the direction of the correlations.

Post-hoc analysis showed that the link between abnormal cortical thickness and the rate of change in psychopathic tendency over time was present in both males than females, with males showing more prominent correlations (Fig 6). Specifically, higher rates of change in psychopathic tendency in males correlated significantly with thicker cortex in the bilateral superior temporal, bilateral middle temporal, left inferior temporal, left isthmus cingulate, left temporal pole, left postcentral and paracentral, bilateral inferior parietal, and bilateral lingual gyri. For females, higher rates of change in psychopathic tendency were found to correlate significantly with thicker cortex in the right superior temporal gyrus, right inferior temporal gyrus, and right inferior parietal lobe.

## Discussion

To our knowledge, this is the first study to show cortical thickness abnormalities associated with individual rates of change in psychopathy over time during childhood and adolescence. Cross-sectionally at age 14, abnormal cortical thickness, particularly thinner prefrontal cortex in females and thicker temporal cortex in males, was associated with elevated psychopathic traits. Furthermore, thicker temporal cortex was linked to higher rates of change in psychopathic tendency between ages 9–18, particularly among males. Our study thus lends support to the concept of psychopathy as a disorder with a neurodevelopmental basis. Overall, this study of healthy community-based adolescents reveals that perturbation of cortical maturation may also play a fundamental role in the development of subclinical levels of psychopathy, rather than simply reflecting comorbid features such as long-term substance abuse or severe delinquent behavior.

We show that cortical thinning in frontal regions, particularly in the right middle frontal gyrus, may be associated with higher psychopathic tendencies in healthy adolescents. This finding is in line with adult finings of cortical thinning in the right middle frontal cortex associated with psychopathy [47]. Thinning in the middle frontal cortex has been associated with behavioral problems including inattention, hyperactive, and impulsive symptoms [48, 49]. Furthermore, the finding of more prominent pathology in the right hemisphere is consistent with prior studies [47, 50] and may be complementary to the finding of increased activation in the right middle frontal gyrus in participants with high psychopathy scores when judging emotionally charged statements and solving moral dilemmas [51–53]. It is important to note that there are few studies on adult offenders that failed to show cortical thickness abnormalities in the middle frontal cortex associated with psychopathy[16, 54]. Thus, findings here suggest that abnormal thinning in the middle frontal cortex may be specific to subclinical levels of psychopathic tendency in relatively healthy samples. Furthermore, findings of stronger correlations between frontal thinning and psychopathic traits in girls are in line with theories [55] that, since the ‘social push’ for psychopathic traits is less prominent in females, a greater degree of neurobiological abnormalities would be required to steer a female in a psychopathic direction.

The somewhat surprising finding of this study was that a *thicker* cortex in several temporal regions was correlated with *not only* concurrent psychopathic traits *but also* individual rates of change in psychopathic tendency over time. We found this to be more prominent in males than females. Although abnormality in the temporal lobe is one of the deficits most frequently linked to psychopathy, thinner cortex in this region is typically observed in adults with psychopathy[16, 47]. This inconsistency may be due to the examination of maturing brains of adolescents. Since cortical thinning including progressive myelination and the systematic ‘sculpting’ of dendritic and synaptic pruning occurs as part of the normal adolescent cortical development, it is possible that a thicker temporal cortex observed here reflects disturbances in this maturation process. Because cortical thickness reflects the proliferation of myelin, findings are also supported by recent findings of disturbed white matter integrity in several frontal and temporal regions including the right superior temporal gyrus to be associated with psychopathic tendency [25, 56–58]. Overall, the abnormally thicker temporal cortex observed here may reflect a lack of refinement in the neural circuits, affecting the supporting cognitive abilities.

The localization of the observed relationship between increasing psychopathic tendencies in adolescent males and thicker temporal cortex, most prominently in the bilateral superior, middle, and the medial temporal gyri (including parahippocampal gyrus, lingual gyrus, and entorhinal gyrus) as well as bilateral temporal poles, may be of significance. These temporal regions work in concert with other frontal and subcortical regions to process information involved in complex social cognition and emotion. Functional abnormalities in these temporal regions have been found in individuals with heightened psychopathic tendencies during emotional face processing [59, 60], semantic processing[61], selective attention[62], and theory of mind [63–65]. Superior temporal cortex, in particularly, is one of the regions most robustly linked to psychopathy. One recently MRI study using pattern classification identified bilateral superior temporal gyri as regions containing the most relevant information for classifying psychopathic individuals [66]. Thus, disturbed cortical development in the temporal lobe, particularly the superior temporal gyrus, may be one of the most promising biomarkers linked to changes in psychopathic tendency among healthy adolescent males.

Last but not least, the left posterior cingulate cortex was also a prominent area where a thicker cortex was associated with an increased propensity for psychopathy. In one recent study, the left posterior cingulate cortex was identified as a brain area most likely to represent an endophenotypes for psychopathy[67]. Specifically, they found that almost half of the genetic variance between increases in psychopathic traits and increases in gray matter concentration in posterior cingulate cortex overlapped, suggesting the development of these two phenotypes to be driven by common underlying genes. The left posterior cingulate cortex is part of the default network involved in empathy, moral judgment, and mentalizing [68–71]. Thus, when the maturation process in this region is disrupted, social and moral functioning may be compromised, as has been observed in psychopathic individuals [72–74]. Although premature, it may be argued that a putative mechanism is at work whereby some genetic variants delay the maturation of this region and result in the worsening of psychopathic symptoms across developmental stages.

This study has several limitations. Although we were able to reduce heterogeneity in brain structural variations within our adolescent sample by matching for age (all were aged 14 at the time of scanning), future studies could benefit from further controlling for pubertal stage. It is also worth noting that findings of this study are based on a community-based healthy adolescent sample with a modest range of parent-reported psychopathy score and no prior substance abuse. Thus, findings may not be generalizable to incarcerated youth with clinical level of psychopathy or substance abuse. We were also unable to identify subtypes of psychopathy such as adolescent limited, childhood limited or life-course persistent due to the modest range of psychopathy scores and the fact that less than 1/3 of the subjects had data from all four waves. Future studies containing a larger adolescent sample with greater range of psychopathy and fewer missing data points would be needed to explore these subtypes of psychopathy. Because the brain scan was only collected at one time point (age 14), we cannot establish that delay in brain maturation contributes to abnormal cortical thickness, resulting in elevated/increasing psychopathy tendency. Lastly, psychopathy scores were available for only 46 subjects at late adolescence (age 16–18), thus our estimation of the rate of change in psychopathy may reflect more closely changes during late childhood and early adolescence in this community sample. Despite these limitations, findings show that abnormal cortical thickness may serve as potential biomarkers for changes in psychopathic tendency during normal development. Multiple brain scans collected in parallel with behavioral and clinical evaluations throughout critical developmental stages in the future will provide the critical information needed to confirm the current findings as well as further our understanding of the neural mechanisms underlying psychopathy in youth and its developmental subtypes.
